# Metabolic bone disease risk factors strongly contributing to long bone and rib fractures during early infancy: A population register study

**DOI:** 10.1371/journal.pone.0208033

**Published:** 2018-12-19

**Authors:** Ulf Högberg, Jacob Andersson, Göran Högberg, Ingemar Thiblin

**Affiliations:** 1 Department of Women’s and Children’s Health, Uppsala University, Uppsala, Sweden; 2 Forensic Medicine, Department of Surgical Sciences, Uppsala University, Uppsala, Sweden; 3 Formerly Department of Women’s and Children’s Health, Child and Adolescent Psychiatric Unit, Karolinska Institutet, Stockholm, Sweden; Medical College of Wisconsin, UNITED STATES

## Abstract

**Background:**

The aim of this study was to assess the incidence of fractures in infancy, overall and by type of fracture, its association with accidents, metabolic bone disease risk factors, and abuse diagnosis.

**Methods:**

The design was a population-based register study in Sweden. Participants: Children born 1997–2014, 0–1 years of age diagnosed with fracture-diagnosis according to International Classification of Diseases (ICD10) were retrieved from the National Patient Register and linked to the Swedish Medical Birth Register and the Death Cause Register. Main outcome measures were fractures of the skull, long bone, clavicle and ribs, categorized by age (younger or older than 6 months), and accident or not.

**Findings:**

The incidence of fractures during infancy was 251 per 100 000 infants (*n* = 4663). Major fracture localisations were long bone (44·9%), skull (31·7%), and clavicle (18·6%), while rib fractures were few (1·4%). Fall accidents were reported among 71·4%. One-third occurred during the first 6 months. Metabolic bone disease risk factors, such as maternal obesity, preterm birth, vitamin D deficiency, rickets, and calcium metabolic disturbances, had increased odds of fractures of long bones and ribs in early infancy (0–6 months): birth 32–36 weeks and long bone fracture [AOR 2·13 (95%CI 1·67–2·93)] and rib fracture [AOR 4·24 (95%CI 1·40–12·8)]. Diagnosis of vitamin D deficiency/rickets/disorders of calcium metabolism had increased odds of long bone fracture [AOR 49·5 (95%CI 18·3–134)] and rib fracture [AOR 617 (95%CI 162–2506)]. Fractures without a reported accident had higher odds of metabolic risk factors than those with reported accidents. Abuse diagnosis was registered in 105 infants, with overrepresentation of preterm births, multiple births and small-for-gestational age.

**Interpretation:**

Metabolic bone disease risk factors are strongly associated with fractures of long bone and ribs in early infancy. Fracture cases with abuse diagnosis had a metabolic bone risk factor profile.

## Introduction

Incidence of fractures during infancy (0–1 year) has been addressed as part of fractures during childhood. Compared to later childhood and adolescence, a lower incidence of fractures is found for infants, 80–326 per 100 000. One reason for this variation might be whether birth-related fractures are included [1–5]. Specific etiologies and risk factors for infant fractures have not been previously addressed [1–5].

The most common cause of fractures during childhood is fall accidents, when Landin’s modified description of trauma level categories of light, moderate, and severe, is applied to define the understanding of clinically evident fractures [1, 5, 6]. In a Swedish study, 8% of the traumatic events among children were either not classified or unknown [1]. The hypothesis that some children might be at higher risk of fractures was raised by Landin, who reports that children having a low bone mineral content can obtain a fracture after a low-energy trauma [7].

Bone strength and stiffness is lower during infancy, being lowest around 4–5 months, compared to adolescents and adults [8]. During the first six months of life, density of the long bones decreases by 30%, in what is called “the physiological osteoporosis of infancy”, however, this is not accompanied by increased bone fragility [9]. Metabolic bone disease includes both excessive bone resorption and impaired bone formation, resulting in reduction in bone mineral content caused by nutritional and biomedical factors [10]. In early infancy, MBD is associated with prematurity, described in observational studies, hospital-based or case reports and reviews, as osteopenia/metabolic bone disease of prematurity [10–12] or temporary brittle bone disease [13]. MBD in preterm-born infants is described to be common between the 10^th^ and 16^th^ week and may range from a silent condition to multiple fractures [10], irrespective of level of trauma [6]. For preterm-born infants, the fracture risk usually stops at the age of 6 months [11]. Rickets and vitamin D deficiency are associated with bone fragility [14–16]. Suggested risk factors include further maternal smoking [17], twinning [18], and small-for-gestational age [19]. Potentially, maternal obesity [20] and ethnicity [21] could contribute to vitamin D deficiency and lower up-take of calcium and phosphorus.

Early bone health is affected by several genetic disorders, such as Osteogenesis imperfecta (OI) [22–24] and Ehlers-Danlos Syndrome (EDS) [22]. Having an improper collagen matrix also suppresses bone formation. [23, 24]. EDS and EDS hypermobility type increases the risk of fractures in adults [25, 26] due to low bone mass and abnormal bone structures [25]. A case series suggested that there is an association between parental Ehlers-Danlos/hypermobility syndrome and multiple fractures in infancy [27]. Genome-wide association studies indicate that pediatric bone mass is largely determined by genetics [22], and this aspect is thus of importance for MBD.A skeletal survey is recommended for the evaluation of suspected infant abuse as there is strong evidence to indicate that injury to the long bone and rib fractures are particularly liable to be due to abuse mechanisms [28]. Occult, clinically silent fractures are detected in 13%–31% of cases surveyed for suspected abuse [29]. Based on hospital studies employing the methodology of determination of abuse by doctors’ interpretation of physical findings, a positive predictive value for abuse has been reported to be 100% for rib fracture [30], and 57% for long bone fracture [31], with an odds ratio of 13·75 for long bone fracture to be indicative of abuse [32]. However, the population incidence of occult fractures is unknown, and these risks might be biased by circular reasoning.

There are several knowledge-gaps regarding fractures during infancy on a population level. To our knowledge, neither incidence nor etiologies, as perceived cause of trauma, abuse, and risk factors of metabolic bone disease, have been addressed in population studies. We hypothesised: 1) if the suggested risk factors for bone fragility are valid, they should be overrepresented in infants having fractures; and 2) the infant age distribution should be pathophysiologically compatible with the suggested bone fragility factors. The aim of this national population register study was to assess the incidence of fractures in infancy, overall and by type of fracture, age, sex, and prematurity, and its association with accidental injury, genetic disorders, abuse diagnosis and metabolic bone disease risk factors.

## Methods

**Fig 1:**
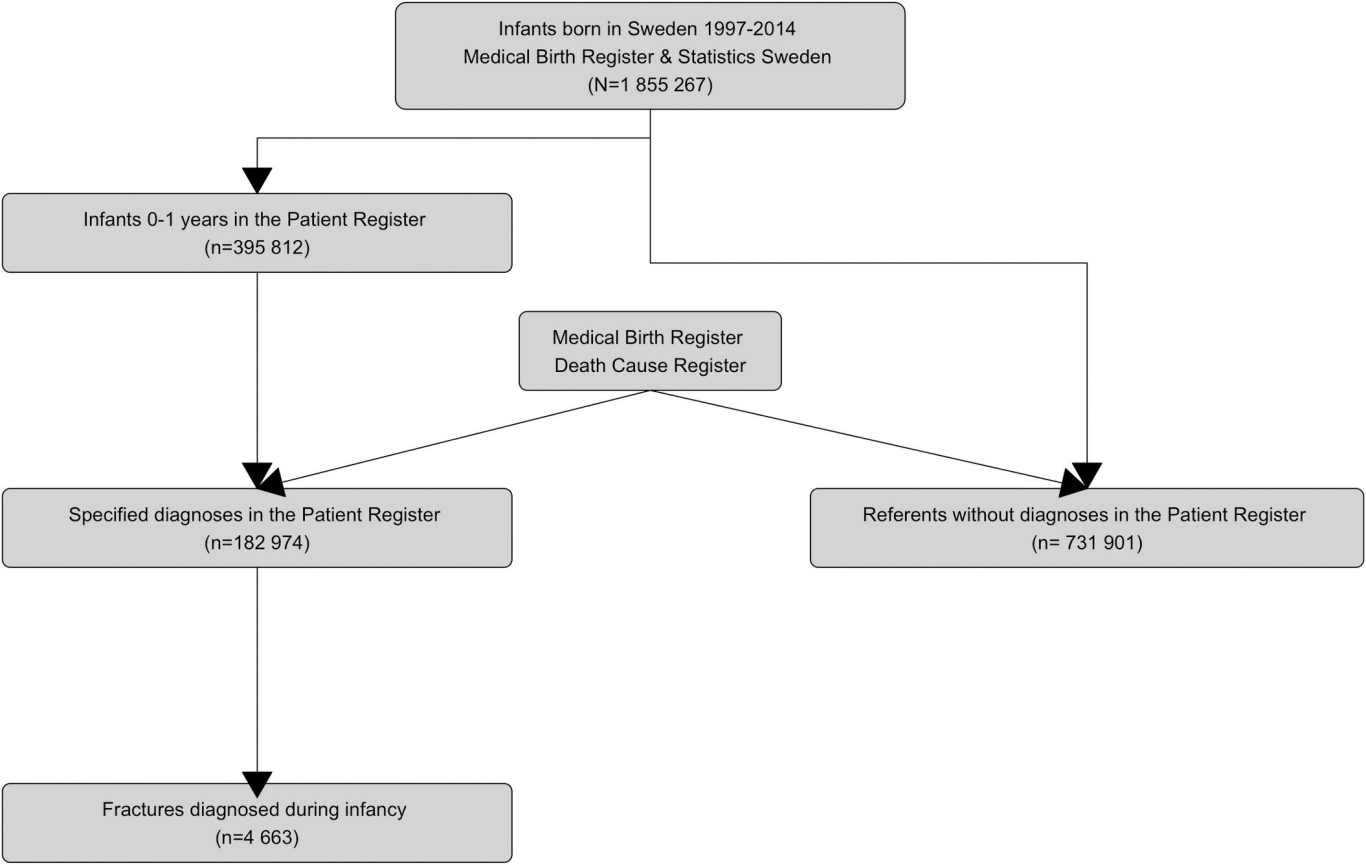
Flow chart of the study base. Source: Patient Register, Medical Birth Register and Death Cause Register, Swedish National Board of Health and Welfare.

This was a nationwide population register study which included infants born in Sweden from 1997 to 2014 with follow-up to one year of age. A flow chart of the data is presented in Fig 1. The source population was infants born in Sweden (*N* = 1 855 267) who had been registered in the National Patient Register (NPR) (*n* = 395 812) [33]. From this, a selection of infants with specified diagnoses were drawn as part of a larger project (*n* = 182 974) [34]. Thus, all registered diagnoses were retrieved from the whole population of infants born during study period. Information from the Medical Birth Register (SMBR) [35] and Cause of Death Register [36] was linked. For analysis of maternal and perinatal factors, four referents, being born in the same year, and without having had any diagnosis registered in the NPR during first year of life, were selected per infant (*n* = 731 901) [34]. Maternal information from the SBMR and NPR were retrieved for the whole study sample. For this study, infants with a selection of fracture diagnoses and accidental injuries, according to the 10th Swedish version of the International Classification of Diseases (ICD10), were identified. Birth-related fractures were excluded. Fall accidents, W00-W19 (ICD10) (Table 1), were classified as: a) slight (<0·5 m)—fall on the same level, fall involving bed, chair or furniture; or b) moderate (>0·5–3m)—fall from being carried and from stairs and steps [1, 5, 6]. Further, for the whole sample, a selection of infant diagnoses, such as osteogenesis imperfecta (OI), Ehlers-Danlos syndrome (EDS) and maternal diagnoses, was extracted (Table 1).

**Table 1:** Definitions of fractures diagnosis, co-morbidity, neonatal morbidity and accidents. 10^th^ revision of the International Statistical Classification of Diseases (Swedish version).

Diagnosis			ICD 10 code
Fractures			
		All^1^	S020, S22, S42, S52, S62, S72
		Skull	S020, S021, S028, S0209 S0200, S029
		Clavicle	S42.0
		Rib	S22.3, S22.4.
		Long bone	S42.2, S42.3, S42,4, S42.7, S42.8, S52, S72, S82
Accidental injury			
	Transport accidents		V01-99
	Fall accidents		W00-19
		From the same level	W01
		While being carried	W04
		Involving bed	W06
		Chair or other furniture	W07/W08
		Playground equipment	W09
		Involving stairs and steps	W010
		From ladder	W011
	Pinch accidents		W23, W52
Maternal diagnoses			
	Preeclampsia		O14
	Ehlers-Danlos/Hypermobility syndrome		Q79.6, M35.7
Infant diagnoses			
	Vitamin D deficiency, Rickets, Disorders of calcium metabolism		E55.9, E55.0, E83.5,
	Osteogenesis imperfecta		Q78.0
	Subdural haemorrhage		I 62.0, S06.5
	Retinal haemorrhage		H356, 362W
	Superficial injury of unspecified body region		T14.0
	Infant abuse diagnosis (observation for suspected abuse, battered baby syndrome, maltreatment syndrome)		Z 03.8K, Y07, T74.1, Y06

^1^Not included: P13 (birth-related), S12 (fracture of neck), S32 (fracture of lumbar spine and pelvis), S62 (fracture wrist hand level), S92 (fracture of foot except ankle)

Outcome measures were fracture of the skull, long bone, clavicle and the ribs with or without transport accidents, or fall accidents. These were categorized by age younger or older than 6 months according to the understanding of bone development during infancy [8, 9], and fracture risk in association with prematurity [11].

Metabolic risk factors of possible importance for fracture risk during infancy following exposure factors were assessed: (1) maternal overweight/obesity defined as BMI at start of pregnancy categorized by overweight (25–29·9) and obesity by class 1 (30–30·9), 2 (35–39·9), and 3 (40+); (2) mother born in Africa, Asia or Latin America; (3) maternal smoking in pregnancy week 30–32 1–9/10- cigarettes; (4) sex; (5) multiple births; (6) preterm birth 32–36/<32; (7) small-for-gestational age (SGA) (<2·5 or <10 percentiles) and SGA Term and Preterm; and (8) infant diagnosis of vitamin D deficiency, Rickets and Disorders of calcium metabolism.

### Statistical analysis

The incidence proportion, or cases per 100 000 infants with 95% confidence intervals (CI), was calculated. Mean, median, and reported accidents were described with descriptive statistics. Mantel-Haenszels chi-square test and Fisher’s exact test were applied to assess differences. Deaths were presented by reported cause of death. Fractures associated with OI and EDS/Hypermobility Syndrome were assessed separately in relation to the study population. In the further analysis of background factors for fractures, OI and EDS were excluded. The distribution of metabolic risk factors, maternal and infant, were displayed for the major fracture groups for the first and second half of the first year. To assess metabolic risk factors for long bone and rib fractures during the first half year of life, crude and adjusted odds ratios, with 95% confidence intervals, were analysed. The statistical software package IBM SPSS 25·0 (SPSS Inc., Chicago, IL, USA) was used for data analyses.

This study was approved by regional ethics committee in Uppsala (2014-11-19 No 383) and was conducted on de-identified data.

## Results

In total, 4 663 fractures were reported in the first year of life in Sweden during 1997–2014, giving an incidence of 2513 per 100 000 infants (Table 2).

**Table 2:** Number of fractures during infancy, by selection of types, incidence per 100 000 (95% confidence intervals), mean and median age, accident and abuse diagnosis in Sweden during the years 1997–2014.

		N (%)	Incidence per 100 000 (CI 95%)	Mean age	Median age	Transport accident (n = 20 091)	Fall accident (n = 41 435)	Pinch accident (n = 782)
				Days	Days	*n* (%)	*n* (%)	*n* (%)
Any fracture		4 663	251·3 (249·0–253·6)	216·9	237·0	208 (4·5%)	3 500 (71·4)	136 (2·8%)
	Skull fracture	1 481(31·7)	79·8 (78·5–81·0)	174·3	178·0	74 (5·0%)	1 242 (83·9%)	3 (0·2%)
	Clavicle fracture	869 (18·6)	46·8 (45·8–47·8)	219·1	238·1	27 (3·1%)	601 (69·2%)	17 (2·0%)
	Rib fracture	66 (1·4)	3·99 (3·70–4·28)	100·3	87·5	6 (9·1%)	17 (25·8%)	1 (1·5%)
Fracture long bones		2 093 (44·9)	112·8 (111·3–114·3)	241·8	272·0	68 (3·3%)	1 507 (72·0%)	75 (3·6%)
	Shaft fracture long bones	458	24·7 (23·9–25·4)	154·8	148·5	22 (4·8%)	306 (66·8%)	29 (6·3%)
	Non-shaft fracture long bone	1 635	88·1 (86·8–89·4)	266·2	294·0	46 (2·8%)	1 169 (71·5%)	46 (2·8%)

### Distribution by type of fracture, age, sex and gestational week

**Fig 2:**
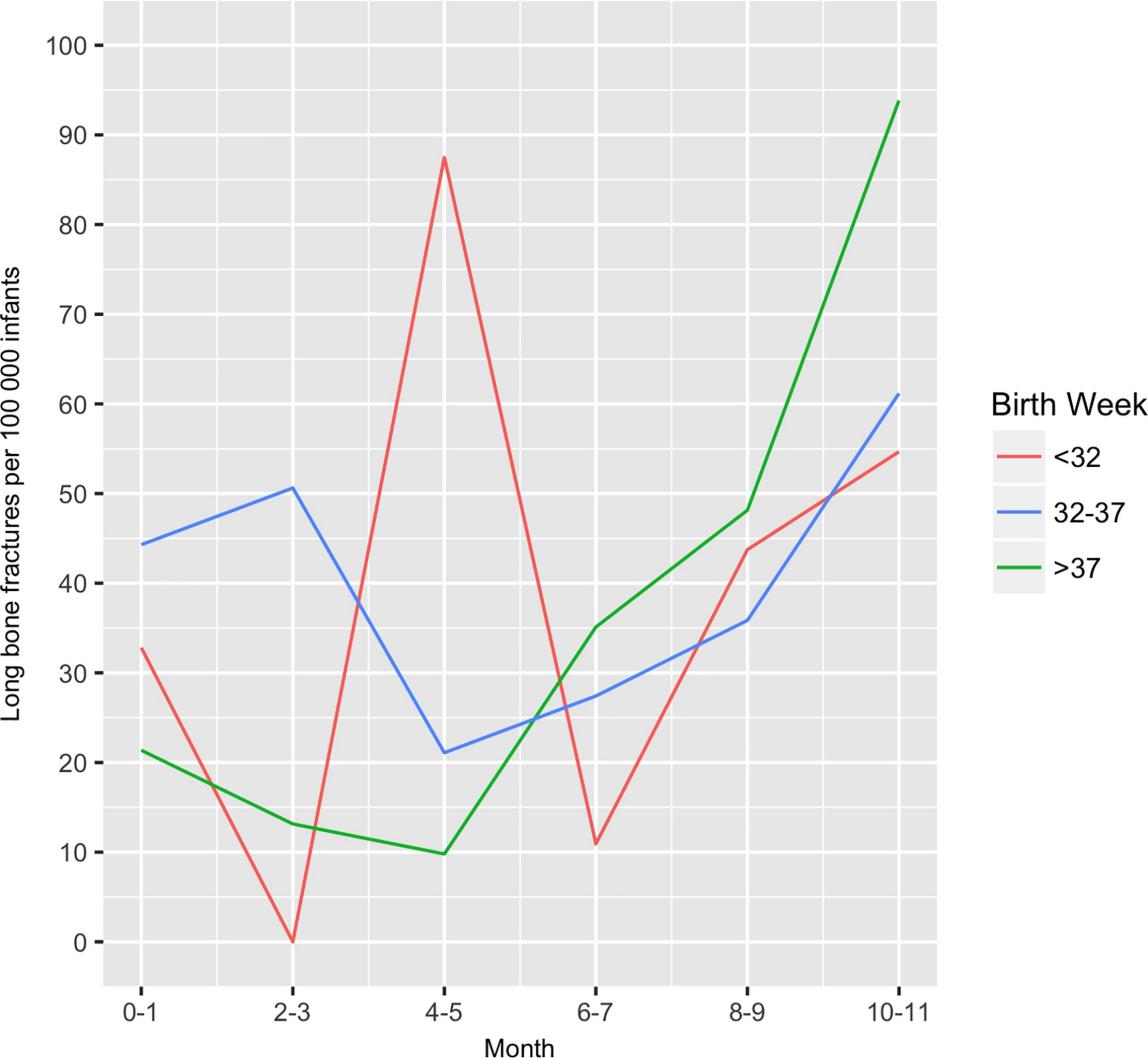
Long bone fractures per 100 000 infants by birth week. Source: Patient Register and Medical Birth Register, Swedish National Board of Health and Welfare.

Almost half of the fractures, 44·9%, were in the long bones, and 31·73% were skull fractures, while only 1·4% had rib fractures (Table 2). Few of the fracture cases had also another category of fracture. For those with a long bone fracture (*n* = 2 093), 19 had fracture of the clavicle, 24 a fracture of the ribs, and 15 a fracture of the skull. However, for those having a rib fracture (*n* = 66), 24 had also had a long bone fracture (32·4%), 13 had a skull fracture (17·6%), and 8 had a clavicle fracture (10·8%). One-third of all occurred within the first six months of life. Mean age at diagnosis for any fracture was 7 months, skull fracture was 5·7 months, shaft fracture of long bone fracture 5·1 months, and for rib fracture, 3·3 months (Table 2). Preterm-born infants were not overrepresented compared to the general population. Preterm-birth infants were at risk of contracting fractures during early infancy, which is shown for long bone fractures in Fig 2. Boys were overrepresented, with 54·1%, compared to 45·9% for girls (*p* = 0·02).

### Accidental injuries

Out of all fractures, 71·4% had a reported fall accident, 4·2% a transport accident, and 2·8% a pinch accident. This pattern was consistent by type of fracture, with the exception of rib fractures, where only 36·4% had a report of an accident (Table 2). Out of the reported fall accidents (*n* = 3 500), 26·6% (*n* = 932) had a slight trauma (fall on the same level, fall involving bed, chair or furniture), and 23·7% (*n* = 831) had a moderate trauma (fall from being carried and from stairs and steps), while the others had unspecified fall accidents.

### Deaths

The cause of death of 54 infants with fractures were: transport accidents (24), diseases (8), malformations (5), perinatal causes (4), falls (3), specified accident events, undetermined intent (5), abuse/homicide (4), and complication of surgery (1).

### Genetic disorders

There were 84 infants with a diagnosis of osteogenesis imperfecta (OI), an incidence of 4·5 per 100 000 infants. Of those with OI, 29 had fractures (long bone 27, skull 2, rib 2, and clavicle 1), out of which 15 had a reported accident, hence, 14 were not reported as being related to an accident. Compared with the study population, infants with OI had an increased risk of contracting a fracture (*p*<0·0000001). There were 511 infants whose mothers had either EDS or EDS/hypermobility syndrome diagnosis, and six of those had fractures (1·2%) compared with the study population (*p* 0·036).

### Fracture and abuse diagnosis

Out of all fracture cases, 105 (2·3%) also had an abuse diagnosis. The distribution by localization of fracture was: skull (33), clavicle (17), ribs (28), shaft long bone fracture (25), and non-shaft long bone fracture (32). Other concomitant diagnoses were subdural haemorrhage (15), retinal haemorrhage (9), and superficial injury (5). A transport accident was reported for 8 infants, 31 had a reported fall accident, and there was one reported pinch accident. Perinatal risk factors in this group were (*p*-value compared to the study population): 21 (20%) preterm-born (*p*<0·0000001), 10 (9·5%) multiple births (*p<*0·0006), and 25 (23·8%) small-for-gestational age <10th percentile (*p*<0·000007). Two infants had a diagnosis of vitamin D deficiency/rickets/calcium metabolic disturbance, and one infant had a diagnosis of osteogenesis imperfecta. Three of the infants with an abuse diagnosis died, and all three had subdural haemorrhage. Other diagnoses were skull fracture (2), clavicle fracture (1), rib fracture (2), and retinal haemorrhage (1). One of these other diagnoses had a transport accident, whereas none had a fall accident.

### Risk factors

Maternal and infant risk factors for long bone fracture and rib facture and age < 6 months or ≥ 6 months of age are presented in Table 3. In relation to risk factors, infants were more prone to contract fractures during the first half year of life. Any fracture at < 6 months and long bone fracture at < 6 months were statistically significant when associated with maternal overweight/obesity, mothers born in Africa, Asia and Latin America, maternal smoking in late pregnancy, preterm birth, and infant diagnosis of vitamin D deficiency/rickets/calcium metabolic disturbances. Long bone fracture was further associated with small-for-gestational age within the < 2·5 and < 10 percentiles. Rib fracture at age < 6 months was statistically significant when associated with multiple births, preterm birth, small-for-gestational-age within the < 10 percentile and infant diagnosis of vitamin D deficiency/rickets/calcium metabolic disturbances. Being born to a mother from Africa, Asia or Latin America was not associated with long bone fracture at < 6 months of age. Vitamin D deficiency/rickets/calcium metabolic disturbances were not associated with fractures beyond 6 months of age.

**Table 3:** Maternal and infant characteristics of infants with fractures categorized by type and age < 6 months and ≥ 6 months during the years 1997–2014 in Sweden. Diagnosis of osteogenesis imperfecta are excluded. Mantel-Haenszel Chi-Square or Fisher’s exact test. *P*-level: a <0·001, b<0·01, c <0·05.

Maternal and infant characteristics		Any fracture		Skull fracture		Long bone		Clavicle		Ribs	
		<6 mths (*n* = 1 551)	≥6 mths (*n* = 3 057)	<6 mths (*n* = 750)	≥6 mths (*n* = 729)	<6 mths (*n* = 504)	≥ 6 mths (*n* = 1 565)	< 6 mths (*n* = 263)	≥ 6 mths (*n* = 605)	<6 mths (*n* = 59)	≥ 6 mths (*n* = 6)
		*n* (*p*)	*n* (*p*)	*n* (*p*)	*n* (*p*)	*n* (*p*)	*n* (*p*)	*n* (*p*)	*n* (*p*)	*n* (*p*)	*n* (*p*)
**BMI**	25–29·9^1^ (*n* = 205 010)	332 (a)	708	145	141	119 (a)	377 (a)	65	160 (c)	13	0
	30–34·9^1^ (*n* = 69 697)	158 (a)	252	65	62	65 (a)	132 (a)	23	44	8	0
	35–39·9^1^ (*n* = 21 318)	46 (a)	67	13	18	29 (a)	29	5	11	2	0
	40+^1^ (*n* = 7654)	11 (a)	26	1 (c)	5	8 (c)	16	1	3	2	0
**Mother’s birthplace**^2^	Africa (*n* = 12 619)	18 (a)	54	7	15	8	22	2	13	0	1
	Asia (*n* = 28 150)	66 (b)	132 (a)	29	31 (c)	21	75 (a)	17 (b)	22	4	0
	Latin America (*n* = 2994)	10 (c)	9	5	4	3	3	1	2	0	0
**Smoking by gestational week**^3^	1–9 (*n* = 34 979) 10+ (*n* = 10 621)	75 (b) 29 (b)	122 36	34 13	30 8	30 9	55 (b) 19	7 8 (b)	25 8	3 0	0 1
**Male**^4^ (*n* = 469 498)		862 (b)	1 619	415 (c)	438 (a)	280 (c)	757 (b)	141	334 (c)	46 (a)	5
**Multiple births**^5^ (*n* = 26 664)		58	76	17	13	30 (a)	49	9	11	6 (b)	1
**Preterm birth**^6^	32–36 (*n* = 47 412)	109 (a)	119 (a)	44	30	54 (a)	59 (c)	5 (c)	23	7 (a)	0
	<32 (*n* = 9146)	29 (a)	18 (c)	11	4	11 (b)	10	1	3	7 (a)	1
**Small-for-gestational age**	<2·5th pctl^7^ (*n* = 20 574)	37	60	21	25	12 (c)	29 (a)	4	0	3	0
	<10th pctl^8^ (*n* = 94 275)	170	285 (c)	88	82	59	137 (c)	18	54	7 (c)	2
**Rickets/DCM/VDD**^9^ (*n* = 353)		13 (a)	2	1	0	8 (a)	2	0	0	5 (a)	0

^1^Reference category: BMI 18·5–24·9 (*n* = 498 493)

^2^Reference category: Scandinavian-born (*n* = 769 965)

^3^Non-smokers (*n* = 775 958)

^4^Reference category: Female (*n* = 439 067)

^5^Reference category: Single births (*n* = 881 907)

^6^Reference category: gestational week 37+ (*n* = 851 572)

^7^Reference category: 881 907

^8^Reference category: 809 965

^9^DCM Disorders of calcium metabolism (DCM)

vitamin D deficiency (VDD), Reference category: 908,201

Presented in Table 4 are crude and adjusted odds ratios of maternal and infant characteristics for long bone and rib fractures at < 6 months of age, categorized by all cases and cases without an accident reported. In general, odds ratios increased for the category without a reported accident, with the exception of male sex for rib fracture, but all odds ratios decreased when adjusted. Maternal overweight/obesity had a trend of increased odds ratios, with obesity class III associated with long bone fracture [AOR 2·48 (95% CI 1·22–5·04)] and rib fracture [AOR 5·67 (95% CI 1·34–24·0)]. Multiple births had an increased crude odds of long bone and rib fractures, but not adjusted. Preterm birth had an increased odds, especially < 32 birth weeks, of long bone fracture [AOR 2·30 (95% CI 1·04–5·07)] and rib fracture [AOR 25·6 (95% CI 6·96–94·4)]. Male sex had increased risk for both long bone and rib fractures. Neither term nor preterm SGA had statistically significantly increased risk of fractures. If an accident was not reported, the odds of long bone and rib fractures were increased for multiple births, and preterm births, and also for SGA, but this was not statistically significant. Diagnosis of vitamin D deficiency, rickets, and calcium metabolic disturbances had the strongest association with fractures; long bone [AOR 49·5 (95% CI 8·3–134)], and rib fractures [AOR 351 (95% CI 99·4–1241)].

**Table 4:** Maternal and infant risk factors for all infants with long bone and rib fractures during the first six month of life, and by selection for those without accidental injury (transport, fall and pinch accidents) reported, born in Sweden during the years 1997–2014. Crude odds ratios (COR), adjusted (AOR) and 95% confidence intervals (95% CI).

		Long bone fracture				Rib fracture			
Maternal and infant risk factors		All (*n =* 504)		Accident not reported (*n* = 188)		All (*n* = 59)		Accident not reported (*n* = 40)	
		COR (95% CI)	AOR^1^	COR (95% CI)	AOR^1^	COR (95% CI)	AOR^1^	COR	AOR^1^
**Overweight obesity** (ref: BMI 18·5–24·9)	25–29·9	1·33 (1·07–1·67)	1·26 (0·99–1·60)	1·30 (0·89–1·88)	1·29 (0·87–1·91)	1·37 (0·70–2·71)	1·31 (0·65–2·65)	0·91 (0·36–2·34)	0·(0·36–2·2·32)
	30–34·9	2·14 (1·63–2·83)	1·92 (1·43–2·59)	2·28 (1·46–3·58)	2·07 (1·27–3·37)	2·49 (1·11–5·56)	1·59 (0·60–4·19)	2·53 (0·83–6·15)	1·32 (0·39–4·54)
	35–35·9	3·13 (2·12–4·61)	2·57´(1·67–3·97)	3·36 (1·96–6·60)	2·82 (1·41–5·67)	2·03 (0·48–8·63)	1·99 (0·47–8·47)	2·96 (0·68–12·9)	2·79 (0·64–12·2)
	40+	2·40 (1·19–4·87)	2·48 (1·22–5·04)	1·67 (0·41–6·77)	1·73 (0·42–7·07)	5·67 (1·34–24·0)	5·39 (1·26–23·06)	4·11 (0·55–31·0)	3·83 (0·51–29·0)
**Smoking w 30–32**	1–9 cig·	1·71 (1·18–2·48)	1·50 (0·99–2·26)	1·29 (0·63–2·63)	1·16 (0·54–2·48)	1·45 (0·45–4·67)	1·08 (0·26–3·4·47)	1·45 (0·35–6·08)	0·84 (0·11–6·20)
	10+	1·69 (0·87–3·28)	1·52 (0·75–3·07)	3·20 ((1·41–7·25)	2·62 (1·07–6·42)	-	-	-	-
**Male** (ref: female)		1·17 (0·98–1·39)	1·29 (1·06–1·58)	1·24 (0·93–1·66)	1·74 (0·83–3·65)	3·31 (1·79–6·13)	3·36 (1·36–8·29)	2·82 (1·38–5·78)	3·36 (1·36–8·29)
**Multiple birth**		2·10 (1·45–3·03)	1·54 (0·97–2·46)	2·73 (1·58–4·70)	1·74 (0·83–3·.65)	3·75 (1·61–8·71)	0·68 (0·15–3·06)	4·84 (1·90–12·4)	0·90 (0·19–4·33)
**Preterm** (ref: 37+)	32–36	2·21 (1·67–2·93)	1·91 (1·33_2·74)	2·35 (1·48–3·74)	2·12 (1·18–3·84)	2·86 (1·29–6·35)	3·68 (1·49–9·10)	3·44 (1·33–8·91)	4·24 (1·40–12·8)
	<32	2·33 (1·28–4·25)	2·30 (1·04–5·07)	2·72 (1·00–7·33)	2·81 (0·82–9·65)	14·83 (6·68–32·9)	16·4 (5·16–51·9)	23·9 (9·90–57·7)	25·6 (6·96–94·4)
**Small–for–** **gestational age (**<2·5th pctl)	Term	0·88 (0·58–1.32)	0·80 (0·33–1·93)	1·44 (0·77–2·70)	1·32 (0·57–3·04)	1·98 (0·49–8·12)	1·76 (0·24–12·9)	2·94 (0·71–12·2)	1·44 (0·33–6·78)
	Preterm	1·67 (0·97–3·88)	0·93 (0·28–3·05))	2·30 (0·86–6·17)	1·23 (0·81–1·88)	3·47 (0·48–25·1)	0·86 (0·10–7·23)	-	-
**Rickets/DCM/VDD**^2^		42·9 (21·4–86·7)	49·5 (18·3–134)	64·1 (23·6–173)	81·7 (20·8–320	243 (96·6–611)	351 (99·4–1241)	325 (115–920)	617 (152–2506)

^1^Adjusted for maternal obesity and smoking, male sex, multiple birth, preterm and small-for-gestational age (<2·5th pctl) term or preterm.

^2^DCM (disorders of calcium metabolism), VDD (vitamin D deficiency)

## Discussion

### Key results

To our knowledge, this is the first population-based study exploring the epidemiology of fracture during infancy and maternal, perinatal and infant risk factors for fractures. This study shows an incidence of studied fractures during infancy of 251 per 100 000 infants. Major fracture localisations were long bone, skull, and clavicle, while few infants had rib fracture. Fall accidents were reported among 3/4 fractures. Bone fragility was evident in association with osteogenesis imperfecta, but was further indicated with Ehlers-Danlos/hypermobility syndrome. One-third occurred during the first 6 months. Metabolic bone disease risk factors, such as maternal obesity, smoking, preterm birth, and vitamin D deficiency, rickets, and disorders of calcium metabolism had increased odds of fractures of long bones and ribs during the infant’s first 6 months. Fractures without a reported accident had higher odds of metabolic risk factors. Those infants having a fracture and abuse diagnosis had an overrepresentation of being preterm-born, multiple-born, and small-for-gestational age in that group.

### Strength and weaknesses of the study

The strength of this study was the population design with national coverage, the prospective data collection, having a uniform 10th version of ICD in place, and probably high reliability of the diagnosis, as a fracture diagnosis requires an x-ray. The dataset, including the referents, contained 49% of all children born in Sweden during the study period (914 875/1 855 267), strengthening its population representativeness. Further, the Swedish health registers are considered to have a high validity [37, 38]. Regarding exposure, a biological association is strengthened by the gradient shown for preterm birth and maternal obesity and odds of fractures. A limitation was that we did not have access to clinical records for further assessment of mechanisms and no possibility to differentiate between symptomatic or occult fractures. In cases of rib fracture, other fractures were present in rather high percentages, indicating that rib fracture might have been occult. Another limitation might be the underreporting of ICD-codes on infants with fractures considered to have been caused by abuse subjected before being taken into out-of-home care by the Social Service. An underestimation of maternal Ehlers-Danlos syndrome/hypermobility syndrome is plausible, as it is mainly diagnosed in primary health care, which is not included in the NPR.

### Interpretation

#### Incidence and accidental injuries

The overall incidence found in this national study was in the lower range of what has been reported from a relatively small hospital-based study (Malmö) [4], and a small cohort in Norway [3]. One reason for the lower incidence in our sample might be that birth-related fractures were excluded, whereas our exclusion of fractures of the neck, lumbar spine, pelvis, wrist, hand and foot should have been of less importance.

In our study, 1/3 of the infants with fractures did not have an ICD-code for accident, while a Swedish hospital-based study had very few cases in which the trauma mechanism could not be determined, namely 4% during 1993–1994, and 0% in 2005–2006 [4]. An explanation for this could be that there were detailed clinical circumstances in the records, but a lack of ICD-code.

#### Genetics

Our results indicate that, in addition to the different genotypes for osteogenesis imperfecta, the parental phenotype of Ehlers-Danlos/hypermobility syndrome should also be investigated. The clinical observations made by Holick et al. [24], indicating a synergetic effect of EDS and vitamin D deficiency for bone fragility and what has been observed in adults with these conditions [22, 23], seems to be in concordance with our results.

#### Risk factors

A main finding of this study was the very high odds of fractures of the long bones and ribs during the infant’s first six months when having a diagnosis of vitamin D deficiency, rickets, or disorders of calcium metabolism. This supports the hypothesis that metabolic bone disease might be one cause for being a fracture-prone infant [1, 11–13]. The bone fragility hypothesis is further supported by our findings of increased odds of metabolic risk factor for fractures without report of an accident. The importance of the intrauterine environments for the risk of fractures during the first six months of life was evident in this study. This association could be a result of lower transfer/loading of micronutrients such as calcium and phosphorus, and prenatal vitamin D deficiency, thus also causing a pathologic bone structure [16] in association with short gestational length, multiple births, and small-for-gestational age, although the latter was not statistically significant in this study. The lack of an association of metabolic bone disease risk factors to fracture during the second six months of infancy could be because the critical period of bone growth occurs during the first six months of life [9, 39], but might also be attributed to the Swedish setting, where children receive efficient child health care and good micronutrient supplementation, including vitamin D, from birth.

This study also adds knowledge of the importance of also mapping the maternal conditions, such as obesity [20] and maternal vitamin D deficiency [14–16], that may cause disturbances in fetal delivery of micronutrients and vitamins, as well as possible malabsorption after bariatric surgery [40] and hyperemesis gravidarum [41], which were not explored in this study.

Several of the risk factors for fractures in infants found in this study, not only extreme prematurity but also moderate preterm and twins, and, further, small-for gestational age, show that it is important that guidelines on the diagnosis of the etiology for infant fractures are not limited to the sampling of well-known biochemical markers for metabolic bone disease and x-rays on the child. Parameters such as parathyroid hormone (PTH), alkaline phosphatase (ALP), ionized calcium, phosphorus, magnesium, and vitamin D may be normal or only slightly affected in a prematurely born infant who has osteopenia with or without signs of rickets at several weeks old [10]. Also, conventional x-ray might have limited value for detecting osteopenia, as a demineralization of more than 20–50% is required [39, 42, 43]. More sensitive methods, have been developed with reference values for newborn and infants, such as Dual energy x-ray absorbitometry (DEXA) [44] and Quantitative ultrasound (QU), providing information about the structure of the bone and about bone density [45], however, further research is needed in their application in clinical practice.

Epidemiological risk factor analysis cannot assert a causal relationship and should be interpreted in relation to bio-mechanical properties of infant bone. There is a scarcity of bio-medical information about infant bone. Ambrose et al. [8] performed mechanical testing of 47 tibia and 52 rib specimen of infant decendants, reporting that strength and stiffness increase by infant’s age, being lowest at around 4–5 months, that girls’ bones have a greater elasticity, while a limited effect of prematurity was seen, although only 4 out of the 9 preterm-born were ≤ 34 gestational weeks. There is a need for more knowledge about the normal maturation of collagen matrix and minerals in early infancy [8] and childhood [46].

#### Fractures and abuse diagnosis

In this study, 105 infants had a fracture diagnosis and abuse diagnosis (in observations of suspected abuse, battered baby syndrome, and maltreatment syndrome), 57 had long bone fractures, and 28 had rib fractures. Within our study design we could not ascertain whether those fracture were clinically overt with symptoms or whether they were occult/silent. Occult fractures are usually detected when a child is investigated for suspected child abuse, which is initiated when an infant has an acute symptomatic fracture, a subdural hematoma or hygroma, or acute deterioration of consciousness associated with some kind of intracranial finding considered to be of traumatic origin, usually a subdural hematoma or hygroma [28]. Occult fractures are sometimes also detected in an asymptomatic sibling who has been examined as part of the abuse investigation.

It might be speculated that the occult fractures detected by skeletal survey for suspected infant abuse in fact reflect metabolic bone disease. The high predictive value for infant abuse, as previously stated [30–32], might be biased by circular reasoning in previous study designs. The systematic review of shaken baby syndrome conducted by the Swedish Agency for Health Technology assessment (SBU) in 2016 went beyond the primary goal of the review and also assessed other systematic reviews, which included long bone and rib fractures and their predictivity in diagnosing abuse [31–32], among others, and found them “to be of low quality (high risk of bias)” due to circular reasoning (p. 11) [47]. The results from our population study support the possibility of bias in hospital studies when the determination of abuse is based on the premise of those fractures being specific to abuse.

### Clinical implications

We suggest that all infants, not only premature ones, having unexplained or occult fractures with no obvious cause in early infancy should be investigated regarding bone fragility as a possible explanation. Our results suggest that in such an investigation the mandatory sampling of biochemical markers for osteopenia and skeletal x-ray investigation need to be complemented with consideration given to all conditions that may affect fetal bone mineralization and that DEXA, or preferable US giving no radiation, should be recommended.

## Conclusion

The incidence of infant fracture is comparable to that previously reported; 2/3 had reported fall accidents. Bone fragility of osteogenesis imperfecta and vitamin D/disorders of calcium metabolism are well known. This study also indicates that bone fragility is associated with Ehlers-Danlos/hypermobility syndrome. Metabolic bone disease risk factors are strongly associated with fractures of the long bone and ribs before 6 months of age, especially without reported accident. The group fracture and abuse diagnosis had a metabolic bone risk factor profile of being preterm born, multiple birth and small-for-gestational age.
